# Multimodal perception-driven decision-making for human-robot interaction: a survey 

**DOI:** 10.3389/frobt.2025.1604472

**Published:** 2025-08-22

**Authors:** Wenzheng Zhao, Kruthika Gangaraju, Fengpei Yuan

**Affiliations:** Department of Robotics Engineering, Worcester Polytechnic Institute, Worcester, MA, United States

**Keywords:** multimodal perception, robot decision-making, human-robot interaction, multimodal fusion, robust autonomy

## Abstract

Multimodal perception is essential for enabling robots to understand and interact with complex environments and human users by integrating diverse sensory data, such as vision, language, and tactile information. This capability plays a crucial role in decision-making in dynamic, complex environments. This survey provides a comprehensive review of advancements in multimodal perception and its integration with decision-making in robotics from year 2004–2024. We systematically summarize existing multimodal perception-driven decision-making (MPDDM) frameworks, highlighting their advantages in dynamic environments and the methodologies employed in human-robot interaction (HRI). Beyond reviewing these frameworks, we analyze key challenges in multimodal perception and decision-making, focusing on technical integration and sensor noise, adaptation, domain generalization, and safety and robustness. Finally, we outline future research directions, emphasizing the need for adaptive multimodal fusion techniques, more efficient learning paradigms, and human-trusted decision-making frameworks to advance the HRI field.

## 1 Introduction

The integration of robots into diverse domains such as healthcare, industrial manufacturing, transportation, and domestic environments has accelerated dramatically in recent years. Across these applications, robots serve various purposes—from providing companionship and assistance to enabling complex collaborations with human users. Despite this diversity of contexts and functions, a fundamental requirement remains consistent: robots must interact appropriately with humans in their specific operational environments. Effective human-robot interaction (HRI) depends critically on a robot’s ability to accurately perceive and understand human users’ status, intentions, and preferences, as well as the surrounding environment, before making appropriate decisions to achieve intended goals. This perception must then inform appropriate decision-making and action planning to achieve specific interaction goals. Consequently, the integration of multimodal perception and decision-making has emerged as a cornerstone of modern HRI research.

However, achieving accurate perception and robust decision-making in HRI remains a significant challenge due to the inherent complexity, dynamism, and variability of human behavior (Amiri et al., 2020), individual preferences, habits, capabilities (Ji et al., 2020), and environments (Diab and Demiris, 2024). Recent advancements in multimodal perception models, such as those leveraging deep learning and large-scale vision-language frameworks (Lu et al., 2024; OpenAI, 2023), coupled with increased computational power, have significantly enhanced robotic capabilities in these areas (Kim et al., 2024; Zhou et al., 2025). These developments have enabled robots to process and fuse data from multiple sensory modalities—such as vision, speech, touch, and proprioception—to form a more comprehensive understanding of their environment and human counterparts. Despite these advancements, the integration of multimodal perception with decision-making frameworks remains an open and actively researched problem, particularly in the context of embodied intelligence for HRI.

While several surveys have explored aspects of HRI, such as multimodal perception (Wang and Feng, 2024), human behavior modeling (Robinson et al., 2023; Reimann et al., 2024), and industrial applications (Duan et al., 2024; Jahanmahin et al., 2022; Bonci et al., 2021), there is a notable gap in the literature. Existing reviews often focus on specific domains, such as manufacturing (Duan et al., 2024; Wang and Feng, 2024; Jahanmahin et al., 2022; Bonci et al., 2021), or narrow aspects of HRI, such as vision (Robinson et al., 2023) or dialogue management (Reimann et al., 2024). To our knowledge, no comprehensive survey has systematically examined the interplay between multimodal perception and decision-making across diverse application domains, including healthcare, manufacturing, and transportation. This gap motivates our work.

In this survey, we present a comprehensive review of over 2 decades of research based on Multimodal Perception-Driven Decision-Making (MPDDM) method in embodied intelligence for HRI. Our primary objective is to analyze how these systems leverage multimodal perception to enable more efficient and accurate decision-making. Specifically, we systematically examine: (1) the sources and types of multimodal sensing data, (2) methodologies for data fusion and perception, (3) decision-making frameworks, and (4) architectures that integrate perception and decision-making. Through this analysis, we identify key challenges and limitations in current approaches and propose potential directions for future research.

Our contributions are threefold:

1. Comprehensive Cross-Domain Coverage: Unlike existing surveys that focus on specific domains, our work synthesizes HRI research across diverse application areas, including industrial manufacturing, healthcare, domestic settings, transportation, and other application areas. This cross-domain perspective provides HRI researchers with a holistic understanding of the current state of technology and methodology in multimodal perception and decision-making, potentially enabling cross-pollination of ideas between domains.
2. Focus on Multimodal Perception: While many reviews emphasize single modalities (e.g., vision or speech), our survey highlights the growing importance of multimodal perception in robotics and HRI. We explore how integrating multiple sensory modalities can enhance perception and decision-making.
3. Integration of Perception and Decision-Making: Our review not only examines multimodal perception but also discusses decision-making frameworks and their integration with perception. This dual focus offers valuable insights for researchers seeking to understand the interplay between these critical components in HRI systems.

By addressing these aspects, our survey aims to serve as a foundational resource for researchers and practitioners in the HRI community, facilitating the development of more robust and context-aware robotic systems.

This survey is structured as follows: Section 2 introduces the study selection process, including database searching, search strategies, and filtering criteria. Section 3 presents the survey findings, discussing the role of multimodal perception in decision-making, strategies for multimodal sensing data fusion, the MPDDM framework, and decision-making methods explored in previous research. Section 4 highlights key challenges, limitations, and potential future research directions in MPDDM within the HRI domain.

## 2 Methodology

### 2.1 Search and selection strategy

**FIGURE 1:**
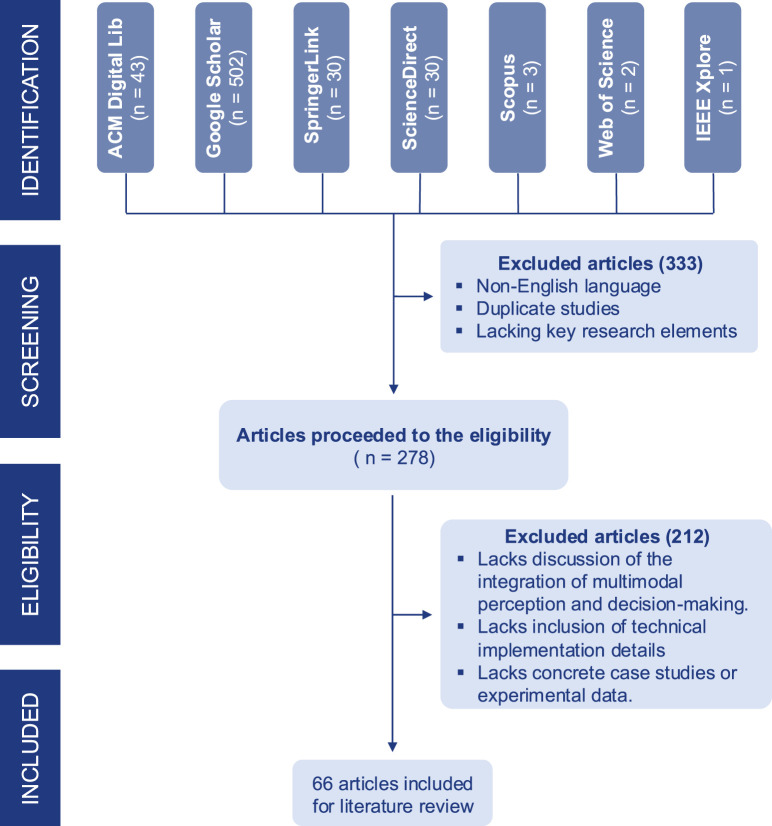
PRISMA flow diagram of the study selection process.

To ensure a comprehensive and systematic review, we followed the Preferred Reporting Items for Systematic Reviews and Meta-Analyses (PRISMA) guidelines (Moher et al., 2009). Figure 1 shows the process of identification, screening, eligibility, and inclusion in this survey. Our search strategy incorporated multiple electronic databases, including Google Scholar, SpringerLink, Web of Science, IEEE Xplore, ScienceDirect, ACM Digital Library, and Scopus, to identify relevant literature on multimodal perception-driven decision-making in human-robot interaction (HRI).

We constructed Boolean search queries based on key terms and their variations to maximize relevant results. The primary search query used in all databases was: (“multimodal perception” OR “multi-modal perception” OR “multisensory perception”) AND “human-robot interaction” AND (“decision-making” OR “decision making”). To refine our search, we applied the studies published from 2004 to 2024.

Using this search query, we obtained 502 hits from Google Scholar, 30 hits from SpringerLink, two hits from Web of Science, one hit from IEEE Xplore, 43 hits from ACM Digital Library, 30 hits from ScienceDirect, and three hits from Scopus. After removing duplicates, 511 articles remained for screening. Upon reviewing the article abstracts and written language, we excluded 233 articles for the following reasons: (1) non-English language, or (2) lacking key research elements for this survey (multimodal perception, human-robot interaction, and decision-making). Thus, 278 articles proceeded to the eligibility review stage. From these, we selected 66 studies that met the following inclusion criteria: (1) detailed work on integrating multimodal perception and decision-making, specifically how multimodal perception aids robots in decision-making for human-robot interaction, (2) inclusion of technical implementation details, including multimodal fusion techniques and perception-driven decision-making methodologies, and (3) concrete case studies or experimental data demonstrating practical human-robot interaction applications.

## 3 Results

To systematically analyze the 66 selected papers following the PRISMA guidelines, we categorized and synthesized each study based on its application domain, multimodal data types, data fusion techniques, and decision-making approaches by leveraging multimodal perception. Specifically, for each paper, we (1) provide a concise summary of its application, (2) identify the types of multimodal data utilized (e.g., vision, audio, language information), (3) classify and analyze the data fusion techniques, distinguishing between Model-Agnostic and Model-Based approaches (see Section 3.3 for details), and (4) examine the decision-making strategies employed (see Section 3.5 for further discussion), with a focus on how multimodal data contributes to improved decision-making performance. A detailed breakdown of each study is presented in Table 3.

### 3.1 Application domains of MPDDM in HRI

Multimodal Perception-Driven Decision-Making (MPDDM) plays a crucial role in various HRI applications. By integrating multimodal perception techniques with decision-making frameworks, robots can operate in dynamic and complex environments with improved adaptability, reliability, robustness, and efficiency. Based on the reviewed literature, MPDDM applications in HRI can be categorized into four primary domains: social and assistive robotics, navigation and mobile robotics, industrial collaboration robotics, and general-purpose robotics with high-level task planning and reasoning. Furthermore, the MPDDM application domains mentioned above exhibit distinct strengths and challenges. Table 1 provides a structured comparison of these domains, highlighting their key advantages as well as limitations and practical challenges, to serve as a reference for future research and application design.

**TABLE 1:** Summary of key advantages and limitations/challenges for major MPDDM application domains.

Application domain	Key advantages	Limitations/Practical challenges
Social and Assistive Robotics	Enhances user engagement, supports emotion recognition and companionship, assists in healthcare and rehabilitation	Sensitive to user diversity (age, culture, cognitive abilities), vulnerable to environmental noise (especially audio), privacy and ethical concerns regarding sensing in healthcare
Navigation and Mobile Robotics	Improves obstacle avoidance and socially-aware navigation, compensates for missing modalities in dynamic environments	High computational load for real-time performance, robustness in crowded/occluded scenarios, variability of human social behaviors across cultures
Industrial Collaborative Robotics	Enhances worker efficiency and safety, enables object manipulation via multimodal attribute learning	Dynamic lighting and environmental variability, human worker behavior unpredictability, cost and complexity of multimodal sensor integration
General-purpose Robotics with High-level Task Planning	Enables robots to understand complex tasks, plan flexible actions across diverse environments, and adapt dynamically based on multimodal perception/feedback	Generalization difficulty to unseen environments, ambiguity in interpreting user intent, computational overhead for multimodal reasoning and real-time adaptation

#### 3.1.1 Social and assistive robotics

Social and assistive robotics are extensively employed in social services, primarily for social interaction, emotion recognition, speech-based dialogue, assistive healthcare, and rehabilitation robotics. These systems aim to enhance user experience and engagement in HRI and provide companion, care, and/or assistance. For instance, previous work designed proactive social robots capable of responding to human emotions (Al-Qaderi and Rad, 2018a), situational states (Vauf et al., 2016), and spatial cues (Ch et al., 2022). Similarly (Tang et al., 2015), developed a companion robot based on a multimodal communication architecture for the elderly. In the field of medical assistance and rehabilitation, researchers explore the potential of MPDDM, for example (Yuan et al., 2024), developed a social robotic framework based on Pepper robot (Pandey and Gelin, 2018) for assisting persons with Alzheimer’s dementia in executing self-care tasks, aiming to enhance their ability to complete daily routines. Additionally (Qin et al., 2023), designed a domestic service interactive robot system, integrating touch, speech, electromyographic gestures, visual gestures, and haptic information, explicitly aiming at individuals with declined expressive abilities.

#### 3.1.2 Navigation and mobile robotics

Autonomous navigation and mobile robotics leverage robotic autonomy and pre-acquired environmental knowledge to facilitate human convenience. For instance, autonomous mobile robots utilizing multimodal perception for obstacle avoidance and navigation (Zhang Y. et al., 2024; Sha, 2024; Wang, 2023; Chen et al., 2020; Roh, 2022; Xie and Dames, 2023) have been extensively studied. These studies have experimentally demonstrated that multimodal perception enhances model robustness, compensating for missing sensory modalities in dynamic environments. Meanwhile, other studies, such as (Panigrahi et al., 2023; Song D. et al., 2024; Siva and Zhang, 2022), focus on socially aware navigation. These works integrate vision, speech, and social signal analysis to enable robots to predict pedestrian trajectories, facilitating socially adaptive and human-friendly navigation strategies.

#### 3.1.3 Industrial collaborative robotics

Industrial collaborative robotics primarily aim to enhance worker efficiency and reduce labor costs by integrating collaborative robots into manufacturing processes. This field includes classic human-robot collaborative assembly tasks (Ji et al., 2020; Forlini et al., 2024; Li et al., 2021; Belcamino et al., 2024) and palletizing robot (Baptista et al., 2024). Other research focuses on leveraging multimodal information to understand and manipulate objects. For instance (Zhang X. et al., 2023), and (Lu et al., 2023) investigate multimodal attribute learning, where robots combine visual, auditory, and haptic data to classify and recognize object properties. Once object attributes are successfully identified, the next challenge is to determine how to grasp and manipulate these objects in dynamic environments. Many researchers adopt Markov Decision Processes (MDP), such as (Amiri et al., 2018) and (Zhang et al., 2021), or reinforcement learning-based models, such as (Balakuntala et al., 2021), to dynamically update robotic actions based on multimodal sensory feedback. More recently, end-to-end learning models, such as (Zhang Z. et al., 2023), have been explored for policy generation in manipulation tasks.

#### 3.1.4 General-purpose robotics with high-level task planning and reasoning

Unlike domain-specific applications, some research focuses on general task planning and decision reasoning across different HRI scenarios. Here, we examine how MPDDM can enable high-level planning beyond single-modal approaches. Traditional task-planning methods in robotics rely heavily on single-modal decision systems. However, in real-world environments, robots encounter uncertainties, dynamic human interactions, and ambiguous sensory inputs, making single-modal task planning insufficient. To address this, previous studies have integrated multimodal sensing data, such as visual, auditory, linguistic, and proprioceptive data, to enhance robotic task planning and situational reasoning. For instance (Forlini et al., 2024; Zhang Z. et al., 2023; Mei et al., 2024), and (Song Y. et al., 2024) leverage GPT/VLM models for semantic task parsing, enabling robots to utilize large-scale vision-language models (VLMs) for end-to-end dynamic task planning. Furthermore, these systems incorporate error correction mechanisms, which allow real-time task adjustments during execution. Beyond predefined task planning, robots operating in unstructured environments must develop situational awareness (Diab and Demiris, 2024), so that robots can adapt their tasks based on real-time environmental states (Amiri et al., 2020; Zhang X. et al., 2024).

### 3.2 Justification of multimodal perception

#### 3.2.1 Multimodal perception

Multimodal perception refers to the study of methods for processing heterogeneous and interconnected data, encompassing both raw signals (e.g., speech, language, images) and abstract concepts (e.g., emotions). By integrating different modalities, humans can better perceive and interpret environmental information. Multimodal perception can be categorized into six primary types: language, vision, touch, acoustic, physiological, and mobile (Liang et al., 2022). Over the past decade, the rapid advancement of deep learning and embodied intelligence has significantly propelled the progress of multimodal perception-driven decision-making. In particular, the emergence of large-scale foundation models such as ChatGPT and Vision-Language-Action (VLA) frameworks has led to a new peak in multimodal development. Currently, most multimodal research focuses on vision and language integration, as researchers aim to enable robots to communicate and interpret the world similarly to humans, leveraging both linguistic reasoning and visual observation to interact with their surroundings. Such cross-modal integration enhances a robot’s ability to comprehend complex scenarios and improves system robustness in the absence of certain sensory inputs. For example, in autonomous driving, if a robot loses radar data in a complex environment, it must still navigate safely using alternative sensory inputs (Camera, etc.) (Grigorescu et al., 2020). Thus, understanding the cognitive processes involved in multimodal data fusion is essential for the future of embodied artificial intelligence.

#### 3.2.2 Advantages of multimodal perception

Single-modal perception (e.g., vision-only, speech-only, or touch-only) has played a role in early research applications. Still, it remains significantly limited in real, complex human-robot interaction scenarios (Huang et al., 2021). The limitations can be delineated as follows: (1) Limited Information: A single sensor provides a restricted perceptual dimension (Wang et al., 2024), making capturing global or deep semantic information challenging. (2) Poor Robustness: Single-modal systems are highly susceptible to noise, lighting changes, occlusions, or hardware failures, leading to performance degradation (Wang et al., 2024). (3) Lack of Accuracy and Generalization: In complex, dynamic environments, single-modal algorithms struggle to maintain high accuracy or adapt quickly (Huang et al., 2021). (4) Inability to Capture Multifaceted Human/Environment Information: Human language, emotions, intentions, and actions involve multiple signals, which a single modality alone cannot fully comprehend (Su et al., 2023).

Due to these limitations, researchers have increasingly focused on multimodal perception (Duncan et al., 2024) in recent years, aiming to integrate information from different types of sensors to handle complex, dynamic HRI scenarios. By integrating different modalities such as text/speech, vision, audio, touch, and physiological signals, multimodal perception offers advantages such as information complementarity and enhanced robustness. For instance (Al-Qaderi and Rad, 2018a), and (Churamani et al., 2020) demonstrated that combining auditory cues with visual inputs improved the accuracy of recognizing personal emotion and location compared to under visual-based detection conditions. Similarly (Granata et al., 2012), leveraged vision and speech to enhance the accuracy and robustness of human detection and interaction in complex scenarios. Furthermore (Wang, 2023), and (Khandelwal et al., 2017) found that multisensory data from both RGB-D cameras and LiDAR sensors mitigated the instability of visual-only systems and improved the robustness of navigation. Beyond robustness, multimodal perception also enhances contextual and semantic comprehension in HRI scenarios. Just like humans, who rely on the integration of multiple modalities (e.g., hearing, vision, smell) to better interpret their surroundings, multimodal perception enables robots to achieve a more comprehensive, accurate understanding of environmental states. For example (Zhang et al., 2021), enabled robots to explore and describe objects in the environment as humans by using audio, haptics, and vision, this approach improved object description accuracy by 50% compared to vision-only exploration.

In summary, multimodal perception not only addresses the inherent weaknesses of unimodal perception but also broadens the scope of MPDDM applications, paving the way for richer human-robot collaboration. However, alongside these advantages, multimodal information also introduces challenges, such as the complexity of data fusion, multimodal representation learning—how to utilize multimodal information effectively, alignment—how to model connections across modalities to ensure accurate understanding and integration, and reasoning—how different modalities interact to influence the decision-making process. These challenges and methods will be explored in Sections 3.3,3.4.

### 3.3 Multimodal sensing data fusion strategies

Multimodal data fusion represents the cornerstone of effective perception-driven decision-making in human-robot interaction systems. This process involves systematically combining information streams from diverse sensing modalities to have a comprehensive representation of the environment. Successful fusion strategies enable robots to overcome the limitations of individual sensors, enhance perceptual robustness in challenging conditions, and develop a more complete understanding of complex human-robot interaction scenarios. In this section, we examine the primary approaches to multimodal data fusion following the framework established by (Baltrušaitis et al., 2018). The fusion methodologies can be broadly categorized into two fundamental classes: model-agnostic methods and model-based methods. Model-agnostic approaches offer flexibility across different learning paradigms, while model-based techniques integrate fusion mechanisms directly within the learning architecture.

Model-agnostic methods typically operate at distinct stages of the perception pipeline, with fusion occurring at the data level (early fusion), feature level (intermediate fusion), decision level (late fusion), or through hybrid combinations spanning multiple processing stages. Meanwhile, model-based methods leverage the inherent capabilities of neural networks, kernel methods, or probabilistic graphical models to learn optimal fusion strategies during the training process. The following subsections detail these approaches, examining their theoretical foundations, implementation considerations, and relative advantages in various HRI contexts.

#### 3.3.1 Model-agnostic methods

Early Fusion (Data-Level): In early fusion, raw or minimally processed data from different modalities are combined into a single input representation at the earliest stage. For example, in a long-term social interaction bartending robot task (Rossi et al., 2024), enhanced the robot’s natural interactive operations by incorporating speech and facial expressions. Similarly (Nan et al., 2019), employed early fusion of RGB and depth images by aligning them based on time frames to improve elderly action recognition. The early-fusion method allows the subsequent model (or pipeline) to learn cross-modal correlations directly from the original data, but it may become challenging to handle large discrepancies or noise across modalities.

Intermediate Fusion (Feature-Level): Features are first extracted independently from each modality, and then these feature representations are fused. This approach balances flexibility and complexity—each modality can be processed separately with tailored feature extraction techniques, and the combined feature space typically captures richer, modality-specific information before final decision-making (Ji et al., 2020). Demonstrated that feature-level fusion of vision, depth, and inertial sensors enables reliable perception by capturing information about humans, robots, and the environment in industrial human-robot collaboration (HRC) (Schmidt-Rohr et al., 2008a). Improved the accuracy of “person of interest” recognition and ensured stable autonomous navigation by converting raw sensory inputs (speech, human activity from RGB-D, and LiDAR) into probability distributions, enhancing dynamic confidence from different feature levels (Scicluna et al., 2024). Showed that aligning 2D feature bounding boxes from RGB with LiDAR depth features prevented false positive detections from leading to incorrect decisions. Similarly, studies such as (Banerjee et al., 2018), (Zhao et al., 2024), and (Deng et al., 2024) enhanced perception capabilities by employing various fusion strategies, including weighting, concatenation, and heuristic-based algorithms.

Late Fusion (Decision-Level): Late fusion focuses on merging the outputs (i.e., decisions or predictions) from multiple models or classifiers. Each modality is modeled separately and the final results are combined (e.g., by voting, averaging, or a learned ensemble). For example (Siqueira et al., 2018), separately predicted emotion and language models, then evaluated the recognition results using a decision framework to resolve emotional mismatches. Similarly (Granata et al., 2013), merged information extracted from four detectors using weighted criteria based on the field of view, reducing the instability of motion prediction when sensor data was incomplete. Late fusion often offers greater robustness if one modality performs poorly, but it may miss certain cross-modal interactions that arise earlier in the data or feature space.

Hybrid Fusion (Combining Multiple Stages): Hybrid fusion integrates multiple fusion strategies. For instance, combining early or intermediate fusion with late fusion. By doing so, it aims to leverage the best of both worlds: capturing cross-modal correlations and ensuring robust final decisions. For example (Sha, 2024), performed feature-level fusion by assigning weights to RGB object detection, depth cameras, and ultrasonic sensors, then integrated obstacle detection and path planning module outputs at the decision level. Similarly (Dean-Leon et al., 2017), applied fusion at multiple stages, including signal level, feature level, and symbolic representation, to enhance collision avoidance, compliance, and grasping strategies. Overall, hybrid fusion approaches aim to leverage the advantages of different fusion stages and integrate them effectively.

#### 3.3.2 Model-based fusion

In this category, the fusion process is driven by a learned model—often nonlinear—such as probabilistic methods, kernel-based methods, neural networks (e.g., CNNs, Transformers), or graph-based models. These approaches learn how to integrate or attend to relevant information across modalities through training, allowing more adaptive and potentially more powerful multimodal representations. For example (Dağlarlı, 2020), utilized probabilistic reasoning and attention mechanisms to integrate multimodal perception data (Zhang et al., 2021). Dynamically constructed a partially observable Markov decision process (POMDP) that integrates information from different sensory modalities and actions to compute the optimal policy.

In the domain of neural network approaches (Yu, 2021), employed CNNs and GANs for gesture/facial synthesis and a hybrid classifier for emotion recognition (Wang, 2023). Utilized an attention mechanism to integrate visual and temporal multimodal features (Yas et al., 2024). extracted RGB and depth data and fused them with skeletal features using a context attention mechanism. Similarly (Al-Qaderi and Rad, 2018b), used spiking neural networks (SNN) to process feature vectors from different modalities, including RGB (FERET), RGB-D (TIDIGITS), and RGB-D (3D body and depth).

Other learning-based approaches include reinforcement learning and graph-based methods (Cuayáhuitl, 2020). Applied deep reinforcement learning (DQN) to fuse visual perception and speech interaction (Ferreira et al., 2012). Employed a graph-based Bayesian hierarchical model to fuse visual and auditory perception. Similarly (Ivaldi et al., 2013), integrated vision, audition, and proprioception using graph-based incremental learning and sensorimotor loops.

More recently, large language models (LLMs) have been leveraged for multimodal fusion (Menezes, 2024). Leveraged, Generative Image-to-text Transformer (GIT) and GPT-4 for cross-modal alignment of visual, textual, and auditory inputs (Forlini et al., 2024). Utilized GPT-4V for feature extraction and decision-making based on visual and contextual inputs. And (Ly et al., 2024) employed an LLM-based planner to generate action sequences by integrating recognition results with motion feasibility.

In summary, multimodal data fusion can be achieved through various strategies—from a simple early fusion of raw data to advanced hybrid and model-based methods that dynamically learn cross-modal interactions. Table 2 summarizes the main fusion strategies discussed above, along with their key advantages and limitations, to provide a concise reference for readers. These approaches provide the foundation for robust and context-rich perception in HRI. In the next section, we explore how these fused representations are integrated into decision-making architectures, enabling robots to leverage the full potential of multimodal inputs for more intelligent and adaptive behavior.

**TABLE 2:** Summary of multimodal fusion strategies, their advantages, and limitations.

Fusion strategy	Advantages	Limitations
Early Fusion (Data-Level)	Enables learning of cross-modal correlations from original data	Sensitive to modality noise, requires careful alignment
Intermediate Fusion (Feature-Level)	Balances flexibility and richer modality-specific information	Dependent on effective feature extraction for all modalities
Late Fusion (Decision-Level)	Robust to failure in individual modalities	May miss useful cross-modal interactions present at earlier stages
Hybrid Fusion	Leverages strengths of different fusion stages	More complex implementation and design effort
Model-Based Fusion	Adaptable and powerful multimodal representations	Typically requires large datasets and can exhibit lower interpretability compared to simpler fusion strategies

### 3.4 Integration architectures for multimodal perception and decision-making

**FIGURE 2:**
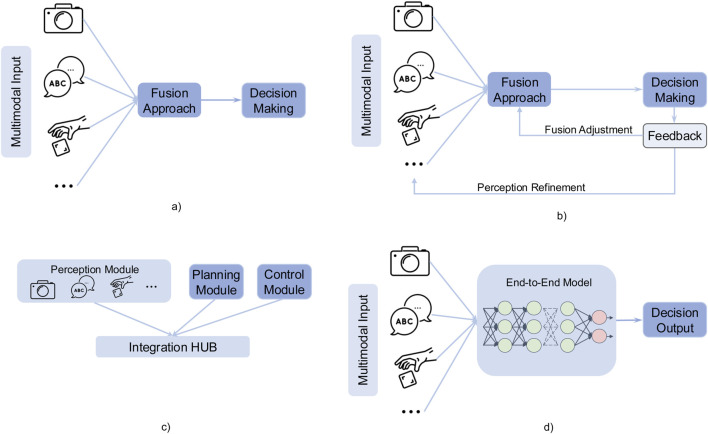
Four basic integration architectures for multimodal perception and decision-making in human-robot interaction. **(a)** Pipeline architecture. **(b)** Feedback architecture. **(c)** Modular architecture. **(d)** End to end architecture.

Multimodal perception can be integrated into decision-making processes for HRI through various architectural frameworks, ranging from conventional linear pipelines to more advanced adaptive models incorporating feedback loops and end-to-end learning. The selection of an appropriate architecture is contingent on multiple factors, including real-time processing constraints, system complexity, and the degree of adaptability required for a given task. For instance, simple feedforward pipelines may be suitable for low-latency applications (Vauf et al., 2016). In contrast, end-to-end frameworks or feedback architecture are often preferred in dynamic and uncertain environments where continuous adaptation is necessary (Mei et al., 2024; Forlini et al., 2024). Therefore, in this section, we analyze the rationale behind the selection of each architectural approach by synthesizing insights from selected papers and empirical findings. We will discuss how different architectures align with specific HRI tasks, the trade-offs they present in performance and adaptability. In multimodal perception-driven decision systems, both academia and industry commonly use five types of high-level architectures to integrate information and execute action decisions for robotics: pipeline architecture, feedback-loop architecture, modular architecture, end-to-end architecture, and hybrid architecture. The first four basic types of architecture have been shown in Figure 2, which illustrates the workflow of each approach.

#### 3.4.1 Pipeline architecture

The pipeline processing architecture allows multiple modalities to be processed simultaneously, reducing processing latency. By handling different sensory inputs in parallel and integrating them through a coordination layer, the system can output multimodal results in real-time, feeding them directly into the planning and decision-making module, as illustrated in Figure 2 (see Subfigure a). This architecture is particularly advantageous in real-time interactive scenarios, where synchronized multimodal processing ensures fast and adaptive responses (Vauf et al., 2016). Implemented a multi-channel parallel processing architecture to detect whether a person intends to initiate interaction with the robot, which enables social companion robots to respond to human behavior more naturally in real-time. They enabled the same robot to acquire and process multiple sensory inputs in parallel, integrating data from different modalities: Laser scanner: 270° field of view, updated every 80 m (12.5Hz), mounted at the base of the Kompaï robot (captures spatial position and distance); Kinect sensor: RGB video (30Hz) for skeleton tracking and facial detection; Depth images (30Hz) for enhanced skeleton tracking; Microphone array: 8Hz for sound source localization and voice activity detection. All features were temporally aligned using an 80 m (12.5Hz) baseline, with data from different modalities fused via temporal synchronization and feature concatenation. The unified multimodal representation was then fed into a classifier (e.g., SVM or neural networks) to recognize interaction intent. They claimed this approach allows the robot to robustly and efficiently infer user engagement, ensuring real-time, natural, and adaptive responses in HRI scenarios.

#### 3.4.2 Feedback-loop architecture

As illustrated in Figure 2 (see Subfigure b), this architecture incorporates feedback mechanisms where outputs from later stages (e.g., decision-making) influence earlier stages (e.g., perception or sensing). This approach enables adaptive and context-aware behavior, improving robustness by allowing the system to refine its perception based on decision outcomes. Such an architecture is particularly well-suited for real-time multimodal perception scenarios in complex human-robot interaction. For example, social robots continuously perceive user emotions and dynamically adjust their dialogue strategies (Ch et al., 2022), or collaborative robots refine grasping operations in real-time using force and visual feedback (Ji et al., 2020; Zhang X. et al., 2023) introduces a pipeline for robotic interaction and perception—the Multimodal Embodied Attribute Learning (MEAL) framework. MEAL enables robots to perceive object attributes—such as color, weight, and empty—through sequential multimodal exploratory behaviors (e.g., observing, lifting, and shaking). The framework is built on a Partially Observable Markov Decision Process (POMDP) for object attribute recognition, structured as follows: Action Selection: The robot selects the next exploratory action (e.g., look, grasp, shake) based on the current environmental state and its belief about the object’s attributes. Information Acquisition: The robot executes the chosen action and collects new sensory data across multiple modalities (e.g., visual, auditory, and tactile features). Belief Update: The system integrates new observations and user feedback (e.g., confirming or correcting attribute recognition) to update the POMDP belief state. In ONLINE-MEAL scenarios, newly collected data (features + labels) are also added to the training set to improve future perception models. Decision Re-evaluation: After updating its belief or model, the system reassesses whether further exploration is needed or if it can confidently report results, forming a closed-loop perception-decision-feedback cycle. Similarly (Zhang Y. et al., 2024), employs multi-source perception (RGB-D, QR codes, wheel odometry, etc.) to obtain the robot’s current state and detect obstacles ahead. The system continuously feeds obstacle location and distance information to the “Safe Manipulation” module in real time. Based on the relative distance and direction between the obstacle and the robot, this module dynamically adjusts the robot’s speed or triggers braking to ensure safe operation.

#### 3.4.3 Modular architecture

The system is divided into independent, self-contained modules, each responsible for a specific function (e.g., sensing, perception, decision-making). Therefore, This architecture is well-suited for scalable robotic systems, particularly industrial collaborative robots. It consists of independent modules, such as a vision detection module (for object localization), a force/tactile sensing module (to ensure safe interaction with humans or objects), a motion planning module (for generating robotic arm trajectories), and a high-level decision-making module (for task allocation and anomaly handling). Each module can be individually upgraded or replaced without affecting the overall system framework. A typical implementation involves clear interfaces or communication protocols between modules, such as topics or services in ROS (Robot Operating System). For instance (Khandelwal et al., 2017), developed a modular and hierarchical general-purpose platform that integrates various independent modules, including mapping, robot actions, task planning, navigation, perception, and multi-robot coordination. Each module has a well-defined function and can be replaced without affecting the overall system. For example, the perception module uses a Kinect camera for human and object detection and LiDAR for environment perception. During real-world operation, the robot continuously perceives its surroundings, updating its knowledge state (via knowledge representation and reasoning nodes) and executing actions based on high-level planning. The key advantages of this architecture include ease of maintenance, upgradability, and flexibility for expanding to more complex tasks. However, a potential drawback is the added system overhead due to module coordination.

#### 3.4.4 End-to-end architecture

End-to-end methods typically involve designing a unified neural network that directly maps sensor inputs to decision-making or action, without requiring manually engineered intermediate steps (Ly et al., 2024). Introduced an “end-to-end” high-level task planning architecture, which processes natural language instructions from humans and integrates visual perception and action feasibility verification. The system combines user input (U), visual observations (O), and feasibility scores (F) into a multimodal context, which is then fed into a fine-tuned Mistral 7B model to automatically generate and execute robot skill sequences (e.g., pick object, move to location, place object), directly mapping to atomic operations from the robot’s existing skill library. Additionally, the framework incorporates failure recovery and a human-in-the-loop mechanism. If the visual perception or feasibility detection module fails, the LLM prompts the user for guidance on handling the failure. The user can then provide new descriptions, suggest alternative objects, or manually reposition objects. The LLM subsequently generates a revised action sequence to ensure task completion. The advantage of this architecture is that it eliminates the need for complex feature engineering and pipeline framework construction. However, its drawbacks include lower interpretability and higher requirements for hardware and algorithms.

#### 3.4.5 Hybrid architecture

The hybrid architecture combines elements from multiple architectural paradigms to leverage their respective strengths. For example, it may incorporate aspects of parallel processing, pipeline structures, and feedback loops. Effectively integrating these mechanisms at different stages or levels can balance real-time performance and flexibility. One example is the brain-inspired multimodal perception system proposed by (Al-Qaderi and Rad, 2018a), which follows a hybrid architecture approach: The system processes different modalities—vision (RGB and depth cameras) and audio (microphones)—in parallel through Dedicated Processing Units (DPUs), each responsible for specialized feature extraction and classification. Within each modality, information flows through a fixed-sequence pipeline, such as facial detection, skeletal tracking, feature extraction, and classification. Moreover, at a higher level, the system integrates spiking neural networks (SNNs) for temporal binding and top-down influences (e.g., using QR codes to infer possible identities), enabling a feedback loop to refine lower-level processing dynamically. This approach allows the system to handle multi-source inputs in parallel, while selectively activating or constraining lower layers based on intermediate recognition results. By doing so, it reduces unnecessary computation and improves response speed. Such a hybrid framework has demonstrated high adaptability and flexibility in dynamic HRI scenarios.

### 3.5 Decision-making methodologies

In the previous subsection, we examined how multimodal perception provides rich context for decision-making by integrating vision, audio, tactile, and other sensory inputs. However, perception alone does not complete the cycle: once the environment is understood, the robot must decide how to act in response (see Section 3.4 for Integration Architectures). Decision-making is a fundamental capability in intelligent systems, enabling robots and AI agents to infer contextual information by perceiving the environment, and then generate appropriate actions. For MPDDM, the system integrates information from multiple sensory modalities—such as vision, audio, touch, and language—to enhance robustness and adaptability in dynamic environments. With the rapid advancement of deep learning, symbolic reasoning, and probabilistic models, decision-making methods have become more adaptive and learning-based paradigms. Table 3 summarizes the decision-making method used by the MPDDM system. However, selecting the appropriate decision-making framework depends on the goal of the task, the level of uncertainty in the environment, and the training data. In this subsection, we summarize the decision-making methodologies that have been studied in HRI, from seven decision-making perspectives. Under each perspective, we presented each method’s distinct strengths and trade-offs in handling environmental uncertainty, data requirements, and the complexity of collaboration:

• Learning-Based Paradigm: Supervised Learning, Reinforcement Learning, Imitation Learning, etc.

• Problem Formulation Based: Markov Decision Process (MDP), Partially Observable MDP (POMDP), Game Theory, etc.

• Symbolic/Logic-Based Approaches: Rule-Based Systems, Automated Planning (STRIPS, HTN), Knowledge Representation, etc.

• Probabilistic Methods: Bayesian Networks, Hidden Markov Models (HMMs), etc.

• Search-Based Planning: Path Planning, Monte Carlo Tree Search (MCTS), etc.

• Generative AI Decision-Making: LLM/VLM-based Decision-Making, Generative Adversarial Imitation Learning (GAIL), etc.

• Hybrid Approaches: Combine multiple methodologies

**TABLE 3:** Summary of multimodal perception and decision-making methods in HRI.

References	Application	Multimodal data	Fusion technique	Decision making process	Decision making method
Schmidt-Rohr et al. (2008b)	Reasoning system for a multi-modal service robot with human-robot interaction	RGB-D; Audio; Self-SLAM; Force + Torque	Bayesian Filtering; Feature Filter; POMDP	POMDP-based decision-making, utilizing fused multimodal belief states for optimal task execution	POMDP
Cai et al. (2024)	Autonomous navigation, intelligent control, and social interaction	RGB-D; Time-Series Data; Audio; Textual Data	Multimodal Data Integration; Attention Mechanisms	Prediction Models (CNN + LSTM + RL)	Supervised Learning
Panigrahi et al. (2023)	Multimodal perception-driven decision-making in social robot navigation	RGB; LiDAR	Outputs of the RNNs from the point cloud and image modules are concatenated and passed through the fusion process	Uses an MLP to generate these waypoints based on multimodal fused features; Predicts linear and angular velocities for immediate motion	Supervised Learning
Dağlarlı (2020)	Social robots that cognitive perception to interact with humans and navigate dynamic environments as personal assistants	Visual; Auditory; Tactile; Laser Range	Probabilistic reasoning and attention mechanisms to integrate multimodal perception data; Linking semantic relations and perception data at the feature level through knowledge anchoring	Dynamic world model integrates multimodal data to facilitate joint attention and cognitive perception to support decision-making (HRI) in social robots	Bayesian Networks
Menezes (2024)	Collaborative target detection involving human operators, virtual robots, and real robots in a mixed virtual-real environment	RGB-D; Auditory; Textual	Use of GIT and GPT-4 for cross-modal alignment of visual and textual/auditory inputs	MuModaR framework integrates multimodal inputs to enable real-time feedback-driven decision-making	LLM-based
Zhang et al. (2023a)	Object-centric learning for robots interacting directly with physical objects to determine their properties	vision, audio, haptics	Hybrid Fusion; Combines multimodal sensory inputs from behaviors into a feature set for attribute identification; Support Vector Machines (SVMs) and weighted feature combinations for modality-specific attribute classification; Observations are aggregated and updated in belief states through probabilistic reasoning (MOMDPs)	MOMDPs handle mixed observability by separating fully observable components (e.g., object grasp success) and partially observable components (e.g., color)	MOMDP
Zhang et al. (2024b)	Navigation system applied in healthcare, logistics, and domestic settings, enhancing the interaction capabilities of mobile robots	RGB-D; QR sensors; Wheel Encoder	Transformers process RGB-D patches for obstacle identification and segmentation; CNNs aggregate multi-layer features for depth estimation; Outputs from QR, RGB-D, and wheel encoders are fused for state estimation and decision-making	Integrates multimodal data for real-time navigation and safe manipulation in complex environments, adapting robot speed and behavior based on perceived obstacles	Rule-based system
Al-Qaderi and Rad (2018a)	Human-Robot Interaction: Implemented in social robots for person recognition, emotion recognition, and context-dependent response	RGB-D (Kinect); RGB camera (USB3 Flea3); microphone (Rode VideoMic); laser rangefinder (SICK LMS-200); sonar sensors	Model-Based Fusion: Liquid State Machines (LSM) and Leaky Integrate-and-Fire (LIF) neurons to perform feature integration	Person recognition using facial, body, and voice features. Adaptive multimodule recognition where missing modalities (e.g., face occlusion) are compensated by available features (e.g., voice)	Supervised Learning
Vauf et al. (2016)	Determining interaction initiation for companion robots based on multimodal perception	RGB-D (Kinect); Microphone (Kinect), Laser Range Finder	Artificial Neural Networks (ANNs) and Support Vector Machines (SVMs) for fusing multimodal features (spatial positions, body postures, and acoustic data) and classifying engagement states	Enhancing decision-making robustness in noisy environments by accurately predicting user interaction initiation intent through integrating multimodal data such as body posture, spatial cues, and audio signals	Supervised Learning
Granata et al. (2012)	Human detection and interaction systems in complex environments	RGB-D (Kinect), Infrared (IR), Laser, Microphone	Independent feature extraction by different detectors (e.g., leg detection via LiDAR, RGB + IR for upper body), with spatial alignment and weighting applied to the features	Confidence values from multimodal fusion guide decisions, ensuring robust detection even under partial occlusion or noise	Rule-based system
Tang et al. (2015)	Human-friendly robot partner to assist the elderly	RGB-D (Kinect); SunSPOT (room brightness, temperature, and human activity logs); Microphone	Spiking neural networks (SNNs) and growing neural gas (GNG) algorithms to process and fuse gesture recognition data	Robot decision-making (e.g., selecting dialogue topics or responding to gestures) is guided by an emotion model computed from multimodal inputs, enabling context-appropriate natural interactions	Reinforcement Learning
Sha (2024)	A novel multimodal perception navigation system for a real open environment	RGB-D (Intel RealSense D456); RGB-IR; 5x Ultrasonic Sensors; GPS; IMU; Audio Data	Features extracted from depth cameras, ultrasonic sensors, and object detection algorithms are weighted and spatially aligned. Outputs from obstacle detection and path planning modules are fused at the decision level based on predefined rules	Achieving comprehensive environmental understanding through multimodal perception with dynamic updates to navigation and interaction strategies	Supervised Learning
Amiri et al. (2018)	Planning for robotic arms using MOMDP with multimodal perception	Visual Data; Haptic Data; Proprioceptive Data; Audio Data; (Thomason16, Sinapov14 Dataset)	Mixed Observability Markov Decision Processes (MOMDPs) are used to integrate multimodal data streams	Modeling fully and partially observable state variables with MOMDP updated through multimodal sensory feedback to optimize subsequent decisions	MOMDP
Ch et al. (2022)	An emotion-driven social robot	RGB (Facial expressions); Speech Data (speech of the users); (Aff-Wild, AFEW-VA, RAVDESS, SAVEE datasets)	MCCNN (Multi-Channel Convolutional Neural Network) extracts and classifies multimodal feature representations	Multimodal perception integrates visual and vocal emotional information, combined with emotional memory and core affect, to enhance negotiation adaptability and user experience	Reinforcement Learning
Ji et al. (2020)	A human-robot collaboration framework using multimodal inputs based on POMDP planning	RGB-D camera; IMUs; Force Sensors	Features from image-based and non-image-based sensors are extracted and combined into unified representations for human behavior recognition and motion planning	The POMDP framework uses multimodal data to update beliefs about the human’s state and intentions, enabling adaptive motion planning	POMDP
Forlini et al. (2024)	A robot-assisted assembly planner based on Large Multimodal Models (LMM)	RGB (2x Intel RealSense D435i); Language (all assembly components, their precedence relationships)	GPT-4V for feature extraction and decision-making based on visual and contextual inputs	Visual and context-aware enables accurate identification of components and their assembly states	VLM-based
Yu (2021)	A social robot for natural human-robot interaction	RGB-D; Thermal cameras; Audio Data	CNN, GANs for gesture/facial synthesis; hybrid classifier for emotion recognition	Enables natural, emotion-sensitive HRI through synchronized multimodal behavior generation	Reinforcement Learning
Wang (2023)	Robust and privacy-preserving autonomous driving	RGB; LiDAR; Radar	ResNet-50 extracts high-level visual features, the LSTM captures temporal dependencies, attention mechanism integrates these multimodal features	Multimodal sensing reduced ambiguity in perception (combining complementary data like RGB and LiDAR) and robustness against single-sensor failures	Supervised Learning
Khandelwal et al. (2017)	A robot for human-robot interaction in open environments	RGB (Kinect); LIDAR (Velodyne); Language	Visual features, spatial maps, and language representations are processed independently and then fused at the decision-making level	Combines probabilistic reasoning and planning (CORPP) to infer missing or ambiguous information, reducing errors in understanding and navigation	Path Planning
Amiri et al. (2020)	A sequential decision-making framework for robots	RGB-D; contextual knowledge	The classifier’s output (probabilistic state estimation) is combined with declarative contextual knowledge using P-log (logical-probabilistic reasoning)	The POMDP planner uses state estimates from sensor data and contextual knowledge as priors to determine the optimal actions for proactive human-robot interaction	POMDP
Zhang et al. (2021)	A multimodal exploratory action interaction robotic arm	RGB; Haptics; Audio	The dynamically constructed POMDP integrates information from different sensory modalities and actions to compute the policy	The decision-making is driven by the POMDP, which integrates multimodal sensory data to refine the robot’s belief state and compute the best action policy	POMDP
Yas et al. (2024)	A multi-agent motion prediction framework	RGB-D; Skeleton Data	Extracts RGB, depth and fuses them with skeletal features using a context attention mechanism	Predicting future trajectories and joint positions of all agents in the scene	Supervised Learning
Diab and Demiris (2024)	An assistive HRI robot for daily life scenarios (kitchen)	RGB; Speech data	Constructs a Knowledge Graph (KG) that integrates semantic labels, relationships, and properties derived from multimodal data	Using object detection and task goal identification, the robot understands the context and adapts actions to collaborate with human preferences	Knowledge representation and reasoning
Li et al. (2021)	A human-robot collaborative assembly (HRCA) robot for aircraft bracket assembly	RGB video; Skeleton data	Intermediate attention captures inter-modality relationships and recalibrates feature maps for better alignment	Recognizing actions and predicting intentions based on visual details and spatiotemporal skeleton data to assist human operators in assembly tasks proactively	Rule-based system
Belcamino et al. (2024)	A multimodal perception framework for human-robot collaboration	RGB-D (Baxter’s cameras, Zed2 camera); 4x IMU; Tactaxis Sensor	Outputs from perception modules (vision, touch, IMU) are integrated at the planning stage, with an HTN planner handling task allocation	Assign specific actions to the robot and human based on current states (e.g., object availability, human activity)	Automated planning (NTH)
Schmidt-Rohr et al. (2008a)	A multimodal home service companion robot	RGB-D; Speech (Sphinx4); LiDAR	Raw sensory inputs (speech, human activities, and navigation) are converted into probability distributions	The robot identifies the “person of interest” based on multimodal cues (speech recognition, human activities) and then autonomously navigates as required	POMDP
Baptista et al. (2024)	A human-robot collaborative manufacturing unit	RGB-D (2x cam); Force sensor; LiDAR	CNN is used to predict to fuse the operator’s next action	Finite State Machine (FSM) transitions between different behaviors (fast object manipulation, safe object handling, object handover) based on multimodal inputs	Rule-based system
Ly et al. (2024)	An interactive home robot	RGB-D; Language	The planner (LLM-based) generates action sequences by integrating recognition results with motion feasibility	The mobile manipulator (Toyota HSR) generates action sequences based on user commands	LLM-based
Zhang et al. (2023b)	An interactive assistive robot (home use)	RGB; Language	A fine-tuned GPT integrates visual input (object detection results), language input (dialogue), and action decision output	The system generates a robotic operation plan based on detected objects and human instructions	LLM-based
Churamani et al. (2020)	An affect-driven human-robot interaction robot	RGB (facial); Audio (auditory)	MCCNN network with two independent channels for facial expression recognition and speech emotion recognition, which are then combined into a unified emotional representation	Use reinforcement learning (RL) to train robots for negotiation in the ultimatum game	Reinforcement Learning
Qin et al. (2023)	A service robot interaction system for individuals with impaired expressive abilities	Touch; speech; EMG gestures; visual gestures; haptics	Different modalities are combined and integrated at the decision level	Multimodal fusion to recognize user intent and respond accordingly	Rule-based system
Lu et al. (2023)	A vision-language interactive grasping robot.s	RGB-D; Language (RoboRefIt)	A transformer-based cross-modal attention mechanism to fuse extracted features (image, text)	Locate and interactively grasp objects using vision + text foundation models and point cloud processing	Supervised Learning
Al-Qaderi and Rad (2018b)	A multimodal perception system for character recognition in social robots	RGB (FERET); RGB-D (TIDIGITS); RGB-D (3D body and depth)	Spiking neural networks (SNN) process feature vectors from each modality	Social robot character recognition with dynamic selection of the most reliable method	Supervised Learning
Scicluna et al. (2024)	Robot navigation and hazard avoidance	RGB, LiDAR; IMU	Align 2D bounding boxes extracted from RGB with LiDAR depth data	Multimodal fusion of RGB, event, and LiDAR ensures FP detection does not lead to incorrect decisions	CVFK
Aly (2014)	Robot behavior adapted to human characteristics	RGB; Speech; Text Data	Coupled Hidden Markov Models (CHMMs) are used to model correlations between speech prosody and gesture features	CHMM-driven decisions synthesize synchronized gestures and prosody for naturalistic robot behavior	HMMs
Zhu et al. (2024)	Multimodal emotion recognition simulating the human brain	RGB; Audio; Text Data; (IEMOCAP Dataset)	Pattern fusion with cross-attention mechanism (ACAM)	The cross-attention mechanism for unified multimodal emotion processing effectively utilizes data from different modalities to enhance emotion recognition accuracy	Supervised Learning
Zhou and Wachs (2019)	Assistant nurse robot (providing medical instruments)	Myo armband; Epoc headset sensor; Kinect sensor	Multimodal signals are synchronized at a 20 Hz frequency and concatenated column-wise	The combination of EEG, EMG, body posture, and acoustic features enables early intent recognition, facilitating predictive decision-making	HMMs
Nan et al. (2019)	Human action recognition on a social assistive robot platform	RGB-D pepper (NTU RGB + D Dataset)	Align RGB and depth maps by time frames	Accurate elderly action recognition model based on OpenPose posture estimation module	Supervised Learning
Siqueira et al. (2018)	Effective emotion recognition under multimodal input conditions	RGB; Speech	Emotion and language models make independent predictions, which are then evaluated using a decision framework	Dynamically resolving emotional mismatches by assessing which modality is more dominant or consistent across multiple interactions	Rule-based system
Dağlarlı (2023)	A perception system (game) architecture mimicking human perception	RGB; Audio	Autoencoder used for feature compression	Determining agent actions (escape or fight) through multimodal scene understanding	Reinforcement Learning
Song et al. (2024a)	Socially aware robot navigation using vision-language models (VLM)	RGB; LiDAR; Language	GPT-4V	VLM-based approach enables adaptive and socially aware decision-making (e.g., recognizing stop gestures)	VLM-based
Siva and Zhang (2022)	A method for long-term human following that adapts to ambient lighting variations in real-world environments	RGB-D	Sparse optimization techniques to learn feature weights for each modality	Stable tracking across three different scenarios representing typical lighting variations	MDP
Ferreira et al. (2012)	Hierarchical Bayesian framework for multimodal active humanoid perception	RGB; Audio	Bayesian hierarchical model integrates visual and auditory perception	Bayesian inference mechanism dynamically adjusts perceptual weights of vision and hearing	Bayesian Inference
Dean-Leon et al. (2017)	A bimanual wheeled humanoid robot with multimodal tactile sensing	RGB; Tactile Skin Sensors; LiDAR	Integration at signal, feature, and symbol levels	TOMM integrates tactile skin input with RGB perception to enhance collision avoidance, compliance, and grasping strategies	Symbolic inference
Pequeño-Zurro et al. (2022)	A welfare robot guidance system	RGB-D; Lidar; IMU	Human key points and corresponding depth maps are projected into 3D space	Vision + depth data estimate human position and velocity, while LiDAR + odometry + IMU enable safe navigation	Supervised Learning
Wu et al. (2019)	Robust robotic arm system under external disturbances	Force-torque sensor; Tactile Sensor; RGB-D; Proprioception	Hierarchical Dirichlet Process Hidden Markov Model (HDP-VAR-HMM) captures underlying patterns of different perception modes and probabilistically learns state transitions for fusion	Fault detection, fault diagnosis, and robot task exploration based on multimodal signals	HMMs
Wang and Feng (2024)	Assistive massage robotic arm	RGB-D; Speech	Features (RGB, depth, gestures, speech, and key points) are extracted separately and then integrated into a classifier for decision-making	Predicting the most likely massage points based on multimodal intent recognition	Bayesian method
Rossi et al. (2024)	Bartending robot for long-term interactive operations	RGB; Speech	Merging detailed data from participating modules (emotion from text semantics, speech, and facial expressions)	Real-time analysis of engagement (posture, speech, emotion), personalized and adaptive robot behavior recommendation (dialogue, gestures), and context-aware beverage preparation	Rule-based systems
Balakuntala et al. (2021)	A robot for performing daily contact-rich tasks (cleaning, writing, and peeling)	RGB-D; Tactile; Force-Torque; Joint Position	CNN for RGB-D, MLP for tactile, force, and joint data, followed by a two-layer MLP for feature integration	Perception outputs are used for trajectory execution (writing, cleaning, peeling) via reinforcement learning algorithms	Reinforcement Learning
Yi et al. (2020)	Smart home service robot	RGB-D; LiDAR; IMU; Audio; Force-Torque	CNN for RGB-D fusion	Path planning and obstacle avoidance, object grasping and picking, speech command interpretation, and user interaction	Supervised Learning
Ivaldi et al. (2013)	Humanoid robot active object learning	RGB-D; Sounds; Proprioception; Force/torque	Integrate visual, auditory, and proprioceptive information using incremental learning and sensorimotor loops	Generate an “interest map” using multimodal sensory data to evaluate different objects and tasks, then determine the most beneficial actions for learning	Reinforcement Learning
Cuayáhuitl (2020)	Interactive multimodal social robot (Tic-Tac-Toe game)	RGB; Speech	Deep reinforcement learning (DQN) model integrates visual perception and speech interaction	The DQN reinforcement learning model integrates multimodal inputs to optimize the robot’s action selection strategy	Reinforcement Learning
Mei et al. (2024)	Autonomous planning robotic arm with error correction mechanism	RGB-D; Language	GPT-4V to learn a joint representation of vision and language data	Multimodal perception to perform robotic task planning with real-time error correction mechanisms	VLM-based
Song et al. (2024b)	Indoor navigation and 3D object recognition based on a multimodal knowledge graph	RGB; Language	CLIP-based encoder extracts embeddings from text and images	Link perceptual (visual) data with conceptual (text) knowledge for contextual reasoning	Knowledge representation and reasoning
Granata et al. (2013)	Human detection and tracking robot	RGB-D; Laser; Microphones	Information extracted from four detectors is merged using weighted criteria based on the field of view	Extended Kalman Filter (EKF) predicts motion when sensor data is incomplete, enabling robust user action tracking	Probabilistic (EKF)
Banerjee et al. (2018)	The robot can perceive the appropriate timing to interrupt human operations	RGB-D	Euclidean distance heuristics to aggregate the output of the various detectors into a single feature vector	Determine when to interrupt humans based on perceived interruptibility	LDCRF
Xie and Dames (2023)	Autonomous navigation robot for spaces with static obstacles and dense pedestrian traffic	RGB (ZED); LiDAR (Hokuyo); Goal point	Two 80 × 80 feature maps from images and one 80 × 80 feature map from LiDAR are combined and fed into a 3 × 3 Conv2D layer	By fusing LiDAR and pedestrian velocity data, the system predicts pedestrian movement patterns, enabling adaptive speed and direction adjustments	Reinforcement Learning
Cohen-Lhyver et al. (2018)	A novel system for regulating robotic exploration behavior	Audio; Visual	M-SOM: where each layer corresponds to audio or video data	The consistency of audiovisual events determines whether to trigger head movement	Knowledge representation and reasoning
Chen et al. (2020)	Audiovisual navigation in a 3D environment	Audio; Visual; GPS	Features from vision, audio, and GPS are fed into a GRU.	When the target is occluded, the agent relies more on audio for localization; when avoiding obstacles, the agent relies more on vision for localization	Reinforcement Learning
Roh (2022)	Autonomous driving robot navigation and human-robot interaction	LiDAR; RGB-D	GraphNet-based fusion, encoding states into a graph	Improve the trajectory prediction using interactive compressed topological representations to enable safe and efficient navigation	Supervised Learning
Jia et al. (2022)	A multimodal emotion recognition model	RGB; Speech; Sequential hand; head action	Three modality features are extracted and fed into an attention layer	Speech (temporal cues), video (spatial facial cues), and motion (body movement cues) to enhance the accuracy and robustness of emotion classification	Supervised Learning
Zhao et al. (2024)	Audiovisual interaction style recognition for children with autism	Video; Audio	Encoders from video and audio are combined through concatenated encoding	Frame-based video features are combined with synchronized audio and speech signals to enhance the understanding of behavior sequences	Supervised Learning
Deng et al. (2024)	Audiovisual recognition of autism-related behaviors	Video; Audio	Use different feature fusion methods, including weighting, max pooling, attention-based weighting, and concatenation	Recognize and classify autism-related behaviors in video segments	VLM-based
Zhang et al. (2024a)	Robot planning in open-world environments	RGB; Language	Pretrained VLM (Vision-Language Model)	When an action fails (e.g., grasping a cup fails or an object drops), the system updates the world state and regenerates the plan	VLM + PDDL
Yuan et al. (2024)	Assistive social robot for Alzheimer’s patients	RGB; Audio; Physiological Signals	Each modality is stored separately, and analysis informs decisions about user performance	Pepper robot provides instructions and evaluates individuals performing ten self-care tasks	Rule-based
Lai et al. (2025)	Service robot framework in aging societies	Voice commands; Deictic posture; RGB-D	Pretrained LLM (GPT4-Turbo) combines verbal commands (action intent) and deictic postures (object selection) to infer complete human intention	Uses GPT-4 to generate task execution sequences, ensuring collision-free movements	LLM-based

#### 3.5.1 Learning-based paradigm

Learning-based decision-making treats the robot (or agent) as a system that acquires policies or value functions from data. Common examples in HRI include supervised learning approaches (e.g., classification, regression), reinforcement learning (RL) for interactive tasks, and imitation learning from human demonstrations. For example, the robot learns to map sensor inputs to discrete actions (e.g., “stop,” “go,” “turn”) based on labeled training sets (Pequeño-Zurro et al., 2022). Similarly, in a collaborative assembly scenario, the robot explores different action strategies, receiving reward signals based on successful or failed assembly interactions (Chen et al., 2025). Alternatively, the robot can observe a human performing a skill and imitate it, adapting its behavior accordingly. For example (Churamani et al., 2020), designed an emotion-driven human-robot interaction system using neural network fusion. Their MCCNN (Multi-Channel Convolutional Neural Network) model consists of two independent channels for facial expression recognition and speech emotion recognition, which are then combined into a unified emotional representation. And then, reinforcement learning (RL) is employed to train the robot on negotiation strategies in the ultimatum game. Similarly (Lu et al., 2023), proposed a vision-language interactive grasping robot, leveraging a transformer-based cross-modal attention mechanism. This system integrates vision, text-based representations, and point cloud processing to enable precise object localization and interactive grasping. Likewise (Al-Qaderi and Rad, 2018b), utilized network-based fusion via a spiking neural network (SNN) to process feature vectors from multiple modalities. This approach enhances multimodal perception for social robots, enabling dynamic and reliable human recognition by selecting the most robust identification method based on the available sensory data. The core advantages of learning-based decision-making include adaptability to new tasks and improving with more data. However, a key drawback is the potentially large data requirement.

#### 3.5.2 Problem formulation based methods

This approach primarily abstracts decision-making as a mathematical model, such as Markov Decision Processes (MDP), Partially Observable MDPs (POMDP), or game theory. Each formulation represents the agent’s state, actions, rewards, and uncertainties. For example (Amiri et al., 2018), employs MOMDPs to integrate multimodal data streams, modeling fully and partially observable state variables with updates from multimodal sensory feedback to optimize future decisions (Amiri et al., 2020). focuses on learning and reasoning for robot sequential decision-making under uncertainty, using a POMDP planner that leverages sensor data and contextual knowledge as priors to determine the optimal action for proactive HRI. Similarly (Zhang et al., 2021), applies a dynamically constructed POMDP to fuse information from different sensory modalities and actions to compute the best policy. The decision-making process is driven by the POMDP framework, which refines the robot’s belief state using multimodal sensory inputs and determines the optimal course of action. This approach’s advantage lies in its clear mathematical framework for problem representation. However, a key drawback is that large state spaces often lead to high computational complexity.

#### 3.5.3 Symbolic/logic-based approaches

This approach relies on symbolic representations (rules, logic programs, knowledge bases) to plan or reason about actions. Examples include rule-based expert systems, automated planning like STRIPS or HTN, and knowledge representation with Answer Set Programming (ASP) or Programming in Logic (Prolog). For example (Diab and Demiris, 2024), designed an assistive HRI robot for daily life scenarios (kitchen tasks), integrating knowledge-based reasoning to support human-robot collaboration. The system utilizes object detection, spatial awareness, and environmental state assessment (e.g., detecting clutter) through a ROS-integrated version of YOLO trained on the COCO dataset. To enable model-based fusion, the framework constructs a Knowledge Graph (KG) that integrates semantic labels, relationships, and properties derived from multimodal data. By combining object detection and task goal identification, the robot understands the scene context and dynamically adapts its actions to align with human preferences, ensuring more intuitive and effective collaboration. This decision-making approach has the advantages of high-level interpretability, accurate capture of domain knowledge, and logically rigorous reasoning. The drawbacks include poor robustness to noise and potential fragility if the rules are incomplete.

#### 3.5.4 Probabilistic methods

This approach primarily uses Bayesian networks, HMMs, factor graphs, or PGM-based methods to model stochastic processes, particularly for uncertainty in human states or the environment. The system continuously updates probabilities as new observations arrive. For example (Dağlarlı, 2020), employs Bayesian networks for cognitive perception, enabling robots to interact with humans and navigate dynamic environments as personal assistants. Similarly (Zhou and Wachs, 2019), utilizes HMMs to integrate EEG, EMG, body posture, and acoustic features, allowing early intent recognition for predictive decision-making. Likewise (Aly, 2014), applies CHMM-driven decision-making to synthesize synchronized gestures and prosody for naturalistic robot behavior. The key advantage of this approach is its principled handling of uncertainty, while the main drawback is the potentially high computational cost for large state spaces.

#### 3.5.5 Search-based planning

Search-Based Planning algorithms (e.g., A*, D*, MCTS) compute a plan or policy by searching the state or action-space. For example (Khandelwal et al., 2017), combines probabilistic reasoning and planning (CORPP) to infer missing or ambiguous information, thereby reducing errors in understanding and navigation. The advantage of this approach is efficient exploration and planning in structured environments, while its main drawback is that it does not handle partial observability or complex uncertainty as effectively as POMDPs.

#### 3.5.6 Generative AI decision-making

Generative AI-based decision-making leverages LLMs or VLMs to generate or refine robot actions. The system can dynamically generate responses and action plans by querying a pretrained GPT-like model with a prompt such as “Given the environment state, what is the next best action?“ (Menezes, 2024).’s MuModaR framework integrates multimodal inputs using GIT and GPT-4 for cross-modal alignment of visual, textual, and auditory inputs, enabling real-time feedback-driven decision-making (Ly et al., 2024). Employs an LLM-based planner to integrate recognition results with motion feasibility, allowing a mobile manipulator (Toyota HSR) to generate action sequences based on user commands (Forlini et al., 2024). Uses GPT-4V to process visual and context-aware inputs, enabling accurate identification of components and their assembly states (Song D. et al., 2024). Developed a socially aware robot navigation system leveraging a VLM-based approach (GPT-4V) to allow adaptive and socially aware decision-making (e.g., recognizing a stop gesture). The advantages of this approach include great flexibility and potential zero/few-shot learning capabilities. While large language models are often perceived as less interpretable than classical rule-based systems, recent techniques such as chain-of-thought (Lu et al., 2024) prompting can improve their transparency and reasoning interpretability. Furthermore, although these models can pose computational challenges in latency-sensitive scenarios, real-time constraints may not be critical in many offline decision-making contexts.

#### 3.5.7 Hybrid approaches

Hybrid Approaches combine two or more methods above—for instance, using symbolic rules with a deep RL agent, or using MDP planning plus an LLM to handle high-level language instruction. This method can exploit complementary strengths, e.g., robust uncertainty handling with interpretability. For example (Zhang X. et al., 2024), developed a robot planning system for open-world environments, leveraging a pre-trained VLM and Planning Domain Definition Language (PDDL) to generate actions. When an action fails (e.g., grasping a cup fails or an object drops), the system updates the world state and replans accordingly. However, integration will be complex.

## 4 Discussion

Building on the previous chapters, we established the necessity of multimodality, highlighted the advantages of multimodal perception in dynamic environments, and summarised how multiple modalities can be fused and integrated into subsequent decision-making processes. We also systematically reviewed the major decision-making methodologies for MPDDM commonly employed in HRI. This section will revisit these findings from a broader perspective, focusing on the current challenges and limitations of existing systems and research efforts. Specifically, we will examine three key areas: technical integration and sensor noise, domain generalization, and safety and robustness. Finally, based on these observations, we propose several future research directions that could guide subsequent investigations and applications in this evolving field.

### 4.1 Current challenges and limitations

#### 4.1.1 Technical integration and sensor noise

In multimodal perception-driven decision-making (MPDDM) systems, the technical integration of sensors and computational modules remains a significant challenge. First, the need to fuse and align data from multiple modalities (e.g., vision, LiDAR, audio) can introduce high computational complexity—the system must handle large-scale data (see (Zhang X. et al., 2023; Al-Qaderi and Rad, 2018a; Zhang Y. et al., 2024; Vauf et al., 2016; Granata et al., 2012; Tang et al., 2015; Sha, 2024; Ji et al., 2020; Wang, 2023; Belcamino et al., 2024)) while ensuring real-time performance. Studies indicate that unimodal perception (using only LiDAR or RGB cameras) is less effective in socially rich or dynamic HRI scenarios (Panigrahi et al., 2023; Zhang Y. et al., 2024; Amiri et al., 2020), and highlight the necessity of incorporating multiple sensors (Cai et al., 2024) for robust situational awareness. However, integrating multiple modalities demands careful calibration, time-stamping, and data synchronization (Sha, 2024; Forlini et al., 2024).

A second challenge relates to computational complexity and real-time constraints. As multiple modalities scale, so do the demands on both memory and processing power, especially when advanced deep learning is used for sensor fusion (Dağlarlı, 2020; Amiri et al., 2018; Granata et al., 2012). This inevitably leads to a significant issue—the need to sacrifice some degree of precision. This trade-off is one of the key reasons why some studies argue that it is difficult to achieve an accurate representation of the real world. For instance, systems that combine raw images, depth maps, and social signals (e.g., speech or gesture data) can overwhelm onboard hardware if not carefully designed (Sha, 2024; Forlini et al., 2024; Ferreira et al., 2012). Consequently, many works struggle to maintain low computational overhead while preserving runtime flexibility and robust decision-making (Forlini et al., 2024; Khandelwal et al., 2017; Zhang et al., 2021; Li et al., 2021). In addition, some works claimed that handling partial observability (e.g., in a mixed-observability MDP) further intensifies the complexity (Amiri et al., 2018; Ji et al., 2020).

Finally, sensor noise and environmental uncertainties remain a pervasive obstacle to reliable MPDDM. Vision modules may suffer inaccuracies from changing illumination or strong reflections, and LiDAR scans can be corrupted by cluttered or reflective surfaces (Zhang Y. et al., 2024; Sha, 2024; Scicluna et al., 2024). Tactile or audio channels can face similar distortions when interacting closely with humans (e.g., voice overlapping in a crowded environment (Zhang Z. et al., 2023; Qin et al., 2023), or haptic signals drowned by mechanical vibration (Forlini et al., 2024)). While some systems attempt to incorporate uncertainty modeling or real-time sensor re-calibration (Li et al., 2021; Al-Qaderi and Rad, 2018b), guaranteeing seamless operation in the presence of sensor noise and incomplete data still remains an open technical challenge.

#### 4.1.2 Domain generalization

Another critical issue for MPDDM in HRI is domain generalization, i.e., whether a trained or engineered system can maintain effectiveness when deployed in new tasks or different application contexts (Zhang X. et al., 2023; Wang, 2023; Baptista et al., 2024; Zhou and Wachs, 2019; Zhang X. et al., 2023). For example, in personal-assistive robots, user demographics and cultural factors significantly affect language or gesture recognition modules (Aly, 2014). Systems that are meticulously tuned to one environment or set of objects often fail to generalize in a new industrial or social setting (Tang et al., 2015; Amiri et al., 2018), leading to increased development costs each time the context changes.

#### 4.1.3 Adaptation

Adaptation is very important in HRI, Despite promising methods for continual learning, many HRI systems still exhibit limited adaptation to changes in user needs or environmental conditions. Trained models may fail when confronted with new geometries, lighting setups, or people’s behaviors (Amiri et al., 2020; Baptista et al., 2024; Churamani et al., 2020; Yuan et al., 2024). Beyond physical changes, the social nature of HRI demands that systems also account for shifting user preferences, habits, cultural norms, and collaborative task requirements, which can evolve over time (Zhang Y. et al., 2024; Granata et al., 2012). Therefore, models optimized for a single/short environment or user profile often become inadequate once real-world conditions diverge from those observed during training. Addressing this challenge calls for robust continual learning strategies that can fuse on-the-fly sensor data with real-time learning/inference while preserving previously acquired knowledge. Developing such flexible adaptation mechanisms remains a key research direction and challenge.

#### 4.1.4 Safety and robustness

Finally, ensuring safety and robustness in real-world HRI scenarios is paramount. Many MPDDM systems must handle close-range human interaction, often in dynamic, unpredictable environments (Menezes, 2024; Granata et al., 2012; Yas et al., 2024). Sensing inaccuracies (e.g., uncertain human motion trajectories or ambiguous gestures) amplify the difficulty of guaranteeing safe robot operation (Tang et al., 2015), particularly when the robot must execute complex manipulation or navigation tasks (Forlini et al., 2024). Although advanced approaches leverage multi-layer sensor fusion and failover mechanisms, long-term deployment can still face drift and sensor misalignment (Menezes, 2024; Granata et al., 2012), leading to cumulative errors over time.

Moreover, real-time failure recovery is frequently overlooked. Some strategies perform global re-planning upon any anomaly, but this can be computationally expensive or slow (Ly et al., 2024). Other works rely on users to intervene manually. The challenge is thus twofold: designing motion-level re-planning or fallback strategies without incurring excessive latency, and building a dialogue or feedback loop allowing humans to provide corrective input (Menezes, 2024; Ly et al., 2024). All these works aim to create a system that not only meets the real-time demands of dynamic HRI but also maintains robust performance and safe collaborative interactions.

### 4.2 Future research directions

#### 4.2.1 Advancing learning-based approaches

Future work would underscore the need for more efficient learning paradigms—ranging from generative AI to reinforcement learning (RL)—to cope with multimodal perception in dynamic HRI. Several works point to semi-supervised or weakly supervised techniques that can reduce reliance on extensive labeled data, thus lowering the costs of large-scale multimodal curation (Cai et al., 2024; Panigrahi et al., 2023; Zhang X. et al., 2023; Sha, 2024). Furthermore, robust generative models could help unify multiple input streams (e.g., vision, audio, haptics) while automatically aligning them with latent representations (Dağlarlı, 2020; Menezes, 2024; Vauf et al., 2016). Equally important is the push to incorporate advanced attention mechanisms and semantic reasoning for better capturing cross-modal signals (Dağlarlı, 2020; Ji et al., 2020; Yas et al., 2024; Aly, 2014), leading to more context-aware and “cognitive” HRI systems. In parallel, scaling up sensor coverage while keeping memory overhead tractable remains a challenge that future work must address by optimizing sensor fusion and feature extraction (Vauf et al., 2016; Belcamino et al., 2024; Scicluna et al., 2024).

#### 4.2.2 Improving explainability and human trust

Ensuring that AI-driven decisions are interpretable is vital for fostering user acceptance and trust in HRI (Mathur et al., 2025). Although many multimodal models yield high accuracy, they often behave as “black boxes,” making it unclear why a system chooses a particular action or how it handles ambiguous inputs (Tang et al., 2015; Qin et al., 2023). Work in (Ch et al., 2022; Li et al., 2021; Zhang Z. et al., 2023) highlights the importance of building affective and dialogue-based interactions with the user, so the robot can clarify uncertainties or explain the reasoning behind certain decisions. Additionally, to promote safer collaboration (Ly et al., 2024; Lu et al., 2023), propose integrated motion-level re-planning frameworks that combine transparency about failure causes (e.g., “object not in view”) with real-time user feedback. Future research might incorporate social cues such as facial expressions, body posture, or personal preferences (Vauf et al., 2016; Churamani et al., 2020; Al-Qaderi and Rad, 2018b; Ferreira et al., 2012; Yuan et al., 2024), improving the system’s ability to provide human-readable justifications and adapt its behavior accordingly. Ultimately, bridging model decisions and intuitive explanations can drive deeper user trust in situations that demand joint decision-making.

#### 4.2.3 Scalable multi-robot collaboration

Another promising direction concerns scalable multi-robot systems, where tasks span collaborative assembly, multi-robot coordination, or large-scale monitoring (Khandelwal et al., 2017; Zhang et al., 2021). While single-robot multimodal perception has progressed substantially, simultaneously coordinating multiple robots under uncertain or partially observable conditions remains underexplored. Key open questions center on robust joint perception—sharing or transferring learned policies, sensorimotor features, and knowledge across heterogeneous platforms (Zhang et al., 2021). In parallel, the complexities of real-world scheduling, path planning, and dynamic role assignment amplify in multi-robot teams, as partial failures in one platform can cascade. Interweaving user interactions—e.g., a human operator or supervisor who provides on-demand clarifications—poses further integration challenges (Khandelwal et al., 2017). Addressing these issues could enable more flexible, self-organized teams of robots that better adapt to large-scale tasks and diverse users.

#### 4.2.4 Long-term autonomy and continual learning

Finally, long-term autonomy in dynamic human environments demands that a robot continuously refine its models and maintain stable performance over lengthy deployments (Zhang Y. et al., 2024; Granata et al., 2012; Amiri et al., 2018; Forlini et al., 2024). Systems must confront persistent changes in environment geometry, lighting conditions, or occupant behavior, which can degrade originally trained models (Amiri et al., 2018). Continual learning approaches that leverage streaming sensor data could keep the robot’s perception and action policies up-to-date—though care must be taken to avoid catastrophic forgetting (Forlini et al., 2024). Equally important is capturing evolving user preferences, social context, and task requirements (Zhang Y. et al., 2024; Granata et al., 2012). Achieving robust online updates for these modules will demand balancing data quality (potentially from incomplete or noisy real-world streams) with computational efficiency, as pointed out by (Amiri et al., 2018) and (Forlini et al., 2024). Future work may combine online transfer learning, policy gradient RL, and environment mapping methods to sustain consistent performance in long-duration, continuously changing settings.

Overall, addressing these four broad directions—advanced learning paradigms, intuitive human trust, multi-robot scaling collaboration, and long-term autonomy—holds the potential to push MPDDM-based HRI toward more natural, capable, adaptable, and user-aligned HRI systems in real-world practice.

## 5 Conclusion

In this survey, we have examined how multimodal perception can enrich decision-making in human-robot interaction from several application perspectives, thereby demonstrating the importance of multimodality in improving decision-making. By synthesizing insights from existing literature, we showed that leveraging multiple sensory modalities not only increases robustness against sensor failures and environmental uncertainties but also provides a richer context for understanding human states and intentions. Consequently, effectively fusing different data modalities into decision models that handle partial observability, real-time constraints, and evolving user behavior has emerged as a critical direction in achieving natural, robust, and safe human-robot interaction in the future.

Despite these promising developments, several challenges remain. Real-world deployments still grapple with sensor noise, synchronization overhead, and the substantial computational burden of processing large-scale multimodal data in real time. Moreover, generalizing systems beyond controlled laboratory conditions poses considerable difficulties—especially when robots operate in diverse settings with varied user profiles, tasks, and cultural norms. Safety and trustworthiness also demand deeper investigation; while fusion-based models achieve higher accuracy, they can be opaque, making it difficult for end users/researchers to understand how a robot arrives at particular choices.

Looking ahead, the ongoing progress of learning-based methods and large-scale foundational models is poised to broaden the horizons of what multimodal perception and decision-making can accomplish. By striking a careful balance among computational efficiency, explainability, and responsiveness, future research can produce truly adaptive, socially aware robots that seamlessly integrate into daily life. Ultimately, overcoming these human-centered challenges will bring us closer to robots capable of robustly perceiving complex scenarios, inferring user intentions and needs, and collaborating safely and intelligently across a wide range of domains.
